# Increased prevalence of myopia in Swedish conscripts between 1975 and 1995 – associations with education and verbal ability

**DOI:** 10.1111/aos.17488

**Published:** 2025-03-19

**Authors:** Tomas Bro

**Affiliations:** ^1^ Department of Biomedical and Clinical Sciences Linköping University Linköping Östergötland Sweden

**Keywords:** cognition, conscripts, education, myopia, prevalence

## Abstract

**Background:**

The objective of this study is to examine the trends in the prevalence of myopia in Swedish young men over a 30‐year period and identify potential risk factors for myopia development.

**Method:**

This retrospective, cross‐sectional study analysed testing results from three cohorts of Swedish military conscripts: 1975, 1985 and 1995. Myopia was defined as a spherical equivalent (SE) of ≤ −0.5 D and high myopia as SE ≤ −5 D. Myopia prevalence was analysed in combination with physical measures (height, weight and BMI), social measures (theoretical upper secondary school) and cognitive measures (4 different abilities). The results from the cognitive tests used a STANdard NINE scale (stanine or S9), a method of scaling test scores on a nine‐point standard scale, with a mean of five and a standard deviation of two.

**Results:**

The study included a total of 13 075 males aged 17 to 19 years. Adequate data on physical measures and vision were available for 95%–98% of participants. The proportion of individuals with myopia increased significantly from 22% to 29% between 1975 and 1995 (*p* < 0.001). High myopia increased from 1.9% to 3.3% (*p* < 0.001). Multivariate logistic regression indicated associations between myopia and year of testing (OR = 1.15), presence of theoretical upper secondary school (OR 1.71, compared to the absence of theoretical upper secondary school), verbal ability (OR 1.08 per stanine) and visuospatial perception (OR 1.05 per stanine).

**Conclusion:**

Over the 30‐year period from 1975 to 1995, the prevalence of myopia among Swedish conscripts increased from 22% to 29%. A higher level of education and higher verbal and visuospatial ability were risk factors for myopia.

## 1 INTRODUCTION

Over recent decades, the World Health Organization (WHO) has reported a concerning rise in myopia. The global prevalence is estimated to have increased from 23% to 28% between 2000 and 2010. The regional differences are considerable, with the highest prevalence numbers in East and Southeast Asia (Holden et al., 2016; WHO, 2017).

Education has long been suspected as a key contributor to the development of myopia. In the late 19th century, an increase in total myopia was observed in central Europe, with the highest prevalence among those with the most education (Cohn, 1883). Meanwhile, in Denmark, Marius Tscherning observed a myopia prevalence of 2% among rural youth compared to 10% among their urban counterparts. The lowest prevalence was noted among sailors and peasants, while the highest rates were found in teachers and veterinarians (Tscherning, 1882). During the 20th century, the focus instead shifted to hereditary causes, and myopia was mainly explained as genetically determined. However, this view was challenged in the early 2000s, as the evidence increased for a rapid, environmentally induced change in the prevalence of myopia, associated with increased education and urbanization (Morgan & Rose, 2005). This development was followed by data supporting that total time outdoors, independent of activity, reduces myopia risk (Jones et al., 2007; Rose et al., 2008). A report in Nature in 2015 marked a significant acceptance of the environmental hypothesis and the epidemic of myopia and high myopia in East and Southeast Asia (Dolgin, 2015). However, the scepticism towards education as a causal risk factor for myopia was not minimized until proven in Mendelian Randomisation studies (Mountjoy et al., 2018). Today, the localization of the Asian epidemic appears to be due to the high educational pressures and limited time outdoors in the region, rather than to genetically elevated sensitivity to these factors (Morgan et al., 2018).

Military recruitment office examinations offer a potential avenue for studying trends in myopia prevalence due to their large participant numbers and standardized methods over extended periods (Table 1). Previous studies on Scandinavian conscripts have reported myopia prevalence in young males, with rates of 13% in Denmark in 2004 (Jacobsen et al., 2007) and 22% in Finland around 2000 (Vannas et al., 2003). The former study employed measured lens power of glasses and standardized subjective refraction, while the latter defined myopia based on self‐reported wearing of ‘minus’ glasses. Comparably higher proportions have been reported in cohorts from Taiwan (Lee et al., 2013) and South Korea (Jung et al., 2012) around 2010, with myopia prevalence rates of 86% and 97%, respectively. Both studies used autorefraction, but only the latter included cycloplegia. Few studies have examined myopia prevalence among conscripts over extended periods. Data from Poland show a stable prevalence around 9% both during 1993–1998 and 1999–2004 (Nowak et al., 2010). In contrast, Austrian data indicate an increase in myopia prevalence (non‐cycloplegic refraction) from 14% to 24% between 1983 and 2017 (Yang et al., 2020), though methodological variations over time may exist. Israeli data on both male and female conscripts showed an increase in prevalence from 20% to 26% between the 1970s and the 1980s (Shapira et al., 2019) and from 20%–28% to 27%–33% between 1990–2002 and 1993–2012 (Bar Dayan et al., 2005; Megreli et al., 2020). Furthermore, the same data also revealed associations between myopia and educational level as well as cognitive testing, particularly verbal ability.

**TABLE 1:** Summary of previous studies of myopia prevalence in military recruitment cohorts.

Author	Country	Year	Age	Myopia
Vannas 2003	Finland	1999–2000	mean 19	22%
Dayan 2005	Israel	2022	16–22	28%
Jakobsen 2007	Denmark	2004	mean 19	13%
Uhlin 2009	Sweden	2022	17–23	38%
Nowak 2010	Poland	1993–2004	mean 22	9%
Jung 2012	Korean	2010	19	97%
Lee 2013	Taiwan	2010–2011	mean 21	86%
Farioli 2017	Sweden	1969–1970	18	13%
Shapira 2019	Israel	1999–2013	mean 17	26%
Thorisdottir 2019	Sweden	1990s	18	29%
Megreli 2020	Israel	1993–2012	mean 17	32%
Yang 2020	Austria	2013–2017	<19	24%

Refractive errors among Swedish conscripts have also been investigated in previous research. Approximately, in 2008, a study involving 650 males found that myopia in the right eye, defined as spherical equivalent (SE) ≤ −0.5 D measured with autorefraction without cycloplegia, was present in 39% of the participants (Uhlin et al., 2009). Additionally, at least two studies have explored refractive data within the Swedish Conscripts Register (SCR). A study investigating the association between rhegmatogenous retinal detachment and occupational lifting exposure reported a myopia prevalence of 13% during 1969–1970, with myopia defined as ≤ −3 dioptres, uni‐ or bilaterality (sphere/SE not described) (Farioli et al., 2017). Another study, conducted with the primary aim of reporting favourable changes in amblyopia prevalence following the initiation of the Swedish visual screening program, noted a significant increase in total myopia, defined as a SE <0 D in the right eye, from 25% to 29% between the 1970s and 1990s (Thorisdottir et al., 2019).

This study aimed to assess the prevalence of myopia among Swedish male conscripts aged 17–19 years from 1975 to 1995, as well as to explore the potential associations of myopia with various risk factors.

## 2 METHODS

Conscription, defined as compulsory enrolment for military service, was mandatory for all young men in Sweden from 1901 to 2010. Until 2007, the testing encompassed over 90% of the corresponding male birth cohorts, involving approximately 45 000 to 55 000 individuals annually from 1975 to 2005. The conscription process, designed to assign individuals to different positions, involved comprehensive testing. Physical measurements had been conducted since the early 1900s, while cognitive testing was introduced in the 1960s (Ludvigsson et al., 2022). This retrospective study utilized data from the Swedish Conscripts Register, incorporating 3781–4630 randomly selected individuals with available data on constitution and vision from three cohorts: 1975, 1985 and 1995 (thus about 10% of the whole total conscription cohort for the selected years).

Recruits were instructed to bring spectacles or eye prescriptions to the visual examination. Corrected and uncorrected visual acuity (Snellen chart at 6 m) in one eye at a time was assessed by nurses trained by opticians. If the uncorrected vision in any eye was below Snellen 1.0, refractive values for spectacles were collected from a lens meter or stated by a prescription. For subjects with contact lenses with no available prescription, refractive values stated by the conscript were noted. In case of reduced visual acuity and absence of spectacles or prescriptions, a non‐cycloplegic manual subjective refraction was conducted with Donders's method (Linroth, 1969; Rendahl, 1972; Thorisdottir et al., 2019). The SE was calculated as the sum of the spherical correction plus half of the cylindrical correction. Total myopia was defined as SE ≤ −0.5 D, and high myopia as SE ≤ −5 D. For the refractive analysis, only data from the right eye were utilized. Eyes without refractive data, but with an uncorrected visual acuity of 1.0, were assumed to have a refractive error of SE ±0. This approach was deemed appropriate to study the distribution of myopia, as myopia is typically absent in eyes with such uncorrected visual acuity (Granstrom, 1952).

The recruits education was categorized in the presence or absence of theoretical upper secondary school. In Sweden, this is an optional three‐year academic pathway after 9 years of compulsory elementary school, leading to the possibility of university entrance. The Swedish school system also has a vocational pathways that lead to vocational qualifications. In this study, this was not regarded as theoretical upper secondary school. The assessment of cognitive abilities comprised four components: logic/general intelligence (inductions), verbal ability (synonyms), visuospatial perception (metal folding) and technical/mechanical skills (technical comprehension). Each subtest included 40 multiple‐choice questions (Carlstedt, 2000). The number of correct answers to these questions was transformed to a discrete variable ranging from 1 to 9, following a Stanine (STAndard NINE) scale that assumes a normal distribution within each year of conscription. Underweight was defined as a BMI lower than 18.5 kg/m^2^.

Statistical significance was assessed using t‐tests for continuous variables and Fisher's exact test for categorical variables. To identify potential risk factors for myopia, a multivariate regression analysis was conducted. Results were presented as odds ratios along with 95% confidence intervals and two‐tailed *p*‐values. Missed values in the regression model were predicted based on the available data with Predictive Mean Matching (PPM), a popular method to generate realistic and plausible imputations (Vink et al., 2014). Approval for the study was granted by the Regional Ethical Review Board in Linköping, Sweden (Dnr 2018–341‐31).

## 3 RESULTS

**FIGURE 1:**
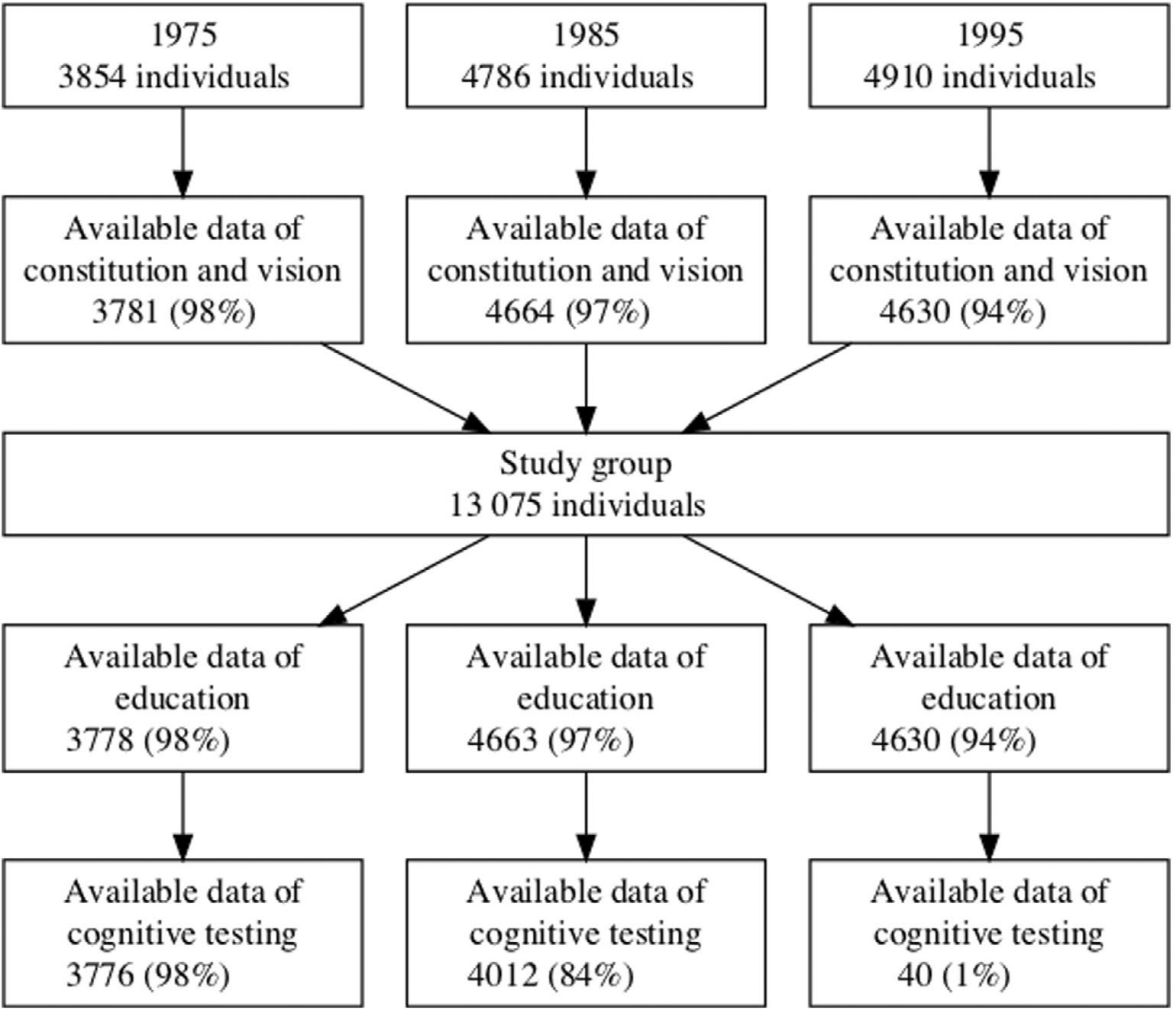
Flow chart of study sample.

**FIGURE 2:**
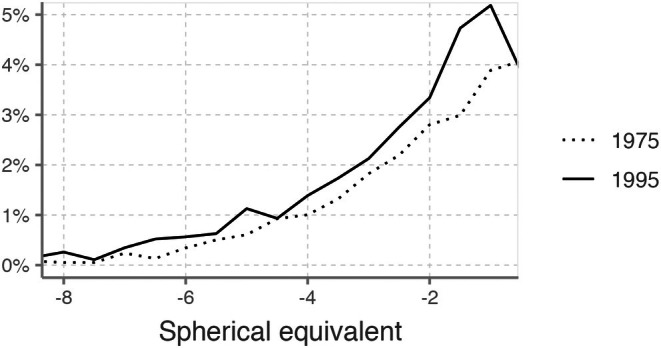
Distribution of spherical equivalent (SE) among myopic men in 2 Swedish military recruitment cohorts 1975 and 1995 (Proportion of the whole population with SE within 0.5 Diopter span. Definition of myopia SE ≤ 0.5 in the right eye.).

A total of 13 075 males aged 17 to 19 were included in the study. Adequate data on constitution, vision and education were available for a substantial proportion (94–98%); however, information from cognitive testing was only available in a reasonable proportion for the first two cohorts (84%–98%) (Figure 1). Over the period from 1975 to 1995, the average height increased by 0.5 cm (from 178.7 to 179.2 cm) (*p* < 0.001). Additionally, there was a significant increase in both average weight (from 68 to 72 kg) and BMI (from 21 to 22) (*p* < 0.001 in both cases). The proportion of individuals free of refractive error (uncorrected visual acuity of 1.0 in both eyes, tested individually) decreased from 69% to 63% (*p* < 0.001). Concurrently, the proportion of individuals with myopia increased from 22% to 29% (*p* < 0.001). When categorized into high myopia, an increase was observed from 0.7% to 1.7%, respectively (*p* < 0.001) (Table 2; Figure 2).

**TABLE 2:** Constitution, education, vision and prevalence of myopia in 3 Swedish military recruitment cohorts between 1975 and 1995.

Characteristic	1975	1985	1995	*p*‐value: 1975/1995
*n*	3781	4664	4630	
Average height in cm (SD)	178.7 (6.6)	179.1 (6.6)	179.2 (6.6)	<0.001
Average weight in kg (SD)	68 (10)	70 (11)	72 (12)	<0.001
Average BMI (SD)	21.4 (2.7)	21.8 (2.9)	22.5 (3.3)	<0.001
Theoretical upper secondary school	33%	47%	46%	<0.001
Uncorrected VA 1.0 in both eyes	69%	66%	63%	<0.001
No myopia (SE > −0.5)	78%	75%	71%	<0.001
Total myopia (SE ≤ −0.5)	22%	25%	29%	<0.001
High myopia (SE ≤ −5.0)	1.9%	1.9%	3.3%	<0.001

Multivariate logistic regression models indicated associations between myopia and several factors. Most importantly, myopia was associated with attainment of theoretical upper secondary school (OR 1.71) followed by the year of testing (OR 1.15), verbal ability (OR 1.08 per stanine) and visuospatial perception (OR 1.05 per stanine) (Table 3).

**TABLE 3:** Multiple logistic regression between factors with possible connections to myopia and myopia prevalence in 3 Swedish military recruitment cohorts between 1975 and 1995.

Group	Characteristic	OR	95% CI	*p*‐value
Myopia total	Year of testing	1.15	1.09, 1.21	<0.001
	Height	1.00	0.99, 1.00	0.3
	Underweight	1.05	0.90, 1.22	0.5
	Theoretical upper secondary school	1.71	1.56, 1.88	<0.001
	Logic/general intelligence	1.00	1.00, 1.01	0.029
	Verbal ability	1.08	1.05, 1.11	<0.001
	Visuospatial perception	1.05	1.03, 1.08	<0.001
	Technical/mechanical skills	1.01	0.98, 1.03	0.6

When comparing myopes to non‐myopes in all tested years, myopes had a significantly higher level of education and higher scores on all cognitive tests. No significant differences could be found in anthropometric values (Table 4).

**TABLE 4:** Comparison of anthropometric, educational and cognitive factors between non‐myopic and myopic men in 3 Swedish military recruitment cohorts between 1975 and 1995.

Characteristic	No myopia	Myopia	*p*‐value
*n*	9701	3347	
Average height in cm (SD)	179.0 (6.6)	179.2 (6.6)	0.091
Average weight in kg (SD)	70 (11)	70 (11)	0.6
Average BMI (SD)	21.9 (3.0)	21.8 (3.0)	0.2
Theoretical upper secondary school	38%	56%	<0.001
Average logic/general intelligence (SD)	5 (14)	6 (6)	0.010
Average verbal ability (SD)	5 (2)	6 (2)	<0.001
Average visuospatial perception (SD)	5 (2)	6 (2)	<0.001
Average technical/mechanical skills (SD)	5 (2)	5 (2)	<0.001

## 4 DISCUSSION

In a population‐based sample of young Swedish men, this study reveals a slight increase in the prevalence of myopia over a two‐decade period (1975–1995). A multiple regression analysis revealed that total myopia was primarily associated with the attainment of theoretical upper secondary school, followed by the year of testing, verbal ability and visuospatial perception. Thus, the rise in education levels emerged as the most significant factor contributing to the increase in myopia.

The findings align with previous studies that have reported a rising trend in the prevalence of myopia among conscripts in the later decades of the 20th century (Bar Dayan et al., 2005; Megreli et al., 2020; Shapira et al., 2019; Yang et al., 2020). It is worth noting that, even if another definition of myopia was used (≤ −0.5 D instead of <0 D), our results are consistent with previously documented refractive changes in Swedish conscripts from the 1970s to the 1990s (Thorisdottir et al., 2019). Several studies have observed that myopic children score higher in cognitive testing (Saw et al., 2004) but to the best of our knowledge, only one previous publication has presented a specific association between myopia and verbal ability (Megreli et al., 2020). As Megreli points out, verbal intelligence requires linguistic skills which are mainly acquired by reading and thus a greater amount of nearwork activity.

The reported myopia prevalence in 1995 of 29% is higher than both data from Denmark (13% in 2004) (Jacobsen et al., 2007) and Finland (22% in 2000) (Vannas et al., 2003). A prevalence of myopia of 39% in Swedish young males in 2009 has been reported in previously published data (Uhlin et al., 2009). However, as the testing of Swedish conscripts only included half of the male population at that time, this result might be affected by a selection bias and not representative of the whole population. The higher level of education and higher score on cognitive testing among myopes in this study correspond well with previous Finnish data (Pärssinen, Era, & Leskinen, 1985; Pärssinen, Leskinen, et al., 1985). However, in the present study, no significant differences could be found regarding BMI.

This study clearly contradicts the previous predictions of a total prevalence of myopia in Western Europe to be 56% by 2050 (Holden et al., 2016). Such an increase could only be achieved if the prevalence of myopia among young adults reaches 80%. Current data indicate a prevalence of 10–30% in Europe, and this study reports an increase of 7% over 20 years. At this rate, it is difficult to envision how a prevalence of 80% could be achieved over the next 30 years.

To the best of our knowledge, this study represents the largest examination of myopia prevalence in Scandinavian young males to date. However, several limitations should be acknowledged. First, the data is almost 30 years old. Initially, a cohort from 2005 was also included, but this group had to be removed as information on constitution and vision was available for less than 50% of the group, and cognitive testing data was entirely missing. Secondly, the study exclusively focused on male recruits, limiting the generalizability of the findings to the broader population. This is crucial as modern data suggest that there is more myopia in females than in males, largely due to a higher level of engagement in education (Enthoven et al., 2024). Third, the retrospective design prevents controls for potential changes in visual measurement practices over the study's time frame. It is therefore unknown if the changes reflect a true difference or a consequence of different methods for data collection. Last but not least, as the measures were performed without cycloplegia, the control of accommodation during the medical investigation is unknown. However, these types of measurement errors are unlikely to bias the analyses on internal trends. This limitation does not undermine the finding of a relatively low myopia prevalence, as cycloplegic examinations would likely further reduce the reported prevalence.

In 2010, the universal conscription system in Sweden was suspended, but due to increased tensions and difficulty attracting recruits, the system was reactivated again by the government in 2017, including both men and women (Ludvigsson et al., 2022). In 2023, 21% of the birth cohort of 2005 is planned to be tested (Plikt‐ och, 2023). Even if the testing is no longer fully representative of the population, it would be interesting in future studies to analyse changes between the tested birth cohorts for the recent years.

Despite several limitations, this retrospective analysis of male Swedish adolescents reveals a notable increase in myopia prevalence between 1975 and 1995 and a significant association with theoretical upper secondary school background and increased verbal and visuospatial abilities.

## FUNDING INFORMATION

This study was funded by Forte – Forskningsrådet för hälsa, arbetsliv och välfärd (Grant number 2019‐00586).

## References

[aos17488-bib-0001] Bar Dayan, Y. , Levin, A. , Morad, Y. , Grotto, I. , Ben‐David, R. , Goldberg, A. et al. (2005) The changing prevalence of myopia in young adults: a 13‐year series of population‐based prevalence surveys. Investigative Ophthalmology & Visual Science, 46, 2760–2765. 16043848 10.1167/iovs.04-0260

[aos17488-bib-0002] Carlstedt, B. (2000) Cognitive abilities ‐ aspects of structure, process and measurement. Göteborg: Acta Universitatis Gothoburgensis.

[aos17488-bib-0003] Cohn, H. (1883) Hygiene des Auges in den Schulen Wien. Leipzig.

[aos17488-bib-0004] Dolgin, E. (2015) The myopia boom. Nature, 519, 276–278. 25788077 10.1038/519276a

[aos17488-bib-0005] Enthoven, C.A. , Haarman, A.E.G. , Swierkowska‐Janc, J. , Tideman, J.W.L. , Polling, J.R. , Raat, H. et al. (2024) Gender issues in myopia: a changing paradigm in generations. European Journal of Epidemiology, 39, 1315–1324. 39661099 10.1007/s10654-024-01163-z

[aos17488-bib-0006] Farioli, A. , Kriebel, D. , Mattioli, S. , Kjellberg, K. & Hemmingsson, T. (2017) Occupational lifting and rhegmatogenous retinal detachment: a follow‐up study of Swedish conscripts. Occupational and Environmental Medicine, 74, 489–495. 28280054 10.1136/oemed-2016-104172 PMC9040060

[aos17488-bib-0007] Granstrom, K.O. (1952) Eyes with normal visual acuity without glasses; an analytical study. Acta Ophthalmologica, 30, 107–114. 12985285

[aos17488-bib-0008] Holden, B.A. , Fricke, T.R. , Wilson, D.A. , Jong, M. , Naidoo, K.S. , Sankaridurg, P. et al. (2016) Global prevalence of myopia and high myopia and temporal trends from 2000 through 2050. Ophthalmology, 123, 1036–1042. 26875007 10.1016/j.ophtha.2016.01.006

[aos17488-bib-0009] Jacobsen, N. , Jensen, H. & Goldschmidt, E. (2007) Prevalence of myopia in Danish conscripts. Acta Ophthalmologica Scandinavia, 85, 165–170. 10.1111/j.1600-0420.2006.00789.x 17305729

[aos17488-bib-0010] Jones, L.A. , Sinnott, L.T. , Mutti, D.O. , Mitchell, G.L. , Moeschberger, M.L. & Zadnik, K. (2007) Parental history of myopia, sports and outdoor activities, and future myopia. Investigative Ophthalmology & Visual Science, 48, 3524–3532. 17652719 10.1167/iovs.06-1118 PMC2871403

[aos17488-bib-0011] Jung, S.‐K. , Lee, J.H. , Kakizaki, H. & Jee, D. (2012) Prevalence of myopia and its association with body stature and educational level in 19‐year‐old male conscripts in Seoul, South Korea. Investigative Ophthalmology & Visual Science, 53, 5579–5583. 22836765 10.1167/iovs.12-10106

[aos17488-bib-0012] Lee, Y.‐Y. , Lo, C.‐T. , Sheu, S.‐J. & Lin, J.L. (2013) What factors are associated with myopia in young adults? A survey study in Taiwan military conscripts. Investigative Ophthalmology & Visual Science, 54, 1026–1033. 23322575 10.1167/iovs.12-10480

[aos17488-bib-0013] Linroth, K. (1969) Medicinska aspekter på ett nytt system för inskrivning av värnpliktiga. Läkartidningen, 66, 881–892. 5794547

[aos17488-bib-0014] Ludvigsson, J.F. , Berglind, D. , Sundquist, K. , Sundström, J. , Tynelius, P. & Neovius, M. (2022) The Swedish military conscription register: opportunities for its use in medical research. European Journal of Epidemiology, 37, 767–777. 35810240 10.1007/s10654-022-00887-0 PMC9329412

[aos17488-bib-0015] Megreli, J. , Barak, A. , Bez, M. , Bez, D. & Levine, H. (2020) Association of Myopia with cognitive function among one million adolescents. BMC Public Health, 20, 647. 32384882 10.1186/s12889-020-08765-8 PMC7206693

[aos17488-bib-0016] Morgan, I. & Rose, K. (2005) How genetic is school myopia? Progress in Retinal and Eye Research, 24, 1–38. 15555525 10.1016/j.preteyeres.2004.06.004

[aos17488-bib-0017] Morgan, I.G. , French, A.N. , Ashby, R.S. , Guo, X. , Ding, X. , He, M. et al. (2018) The epidemics of myopia: Aetiology and prevention. Progress in Retinal and Eye Research, 62, 134–149. 28951126 10.1016/j.preteyeres.2017.09.004

[aos17488-bib-0018] Mountjoy, E. , Davies, N.M. , Plotnikov, D. , Smith, G.D. , Rodriguez, S. , Williams, C.E. et al. (2018) Education and myopia: assessing the direction of causality by mendelian randomisation. BMJ, 361, k2022. 29875094 10.1136/bmj.k2022 PMC5987847

[aos17488-bib-0019] Nowak, M.S. , Jurowski, P. , Gos, R. & Smigielski, J. (2010) Ocular findings among young men: a 12‐year prevalence study of military service in Poland. Acta Ophthalmologica, 88, 535–540. 19456312 10.1111/j.1755-3768.2008.01476.x

[aos17488-bib-0020] Pärssinen, O. , Era, P. & Leskinen, A.L. (1985) Some physiological and psychological characteristics of myopic and non‐myopic young men. Acta Ophthalmologica. Supplement, 173, 85–87. 3002114 10.1111/j.1755-3768.1985.tb06852.x

[aos17488-bib-0021] Pärssinen, O. , Leskinen, A.L. , Era, P. & Heikkinen, E. (1985) Myopia, use of eyes, and living habits among men aged 33‐37 years. Acta Ophthalmologica, 63, 395–400. 4050359 10.1111/j.1755-3768.1985.tb01551.x

[aos17488-bib-0022] Plikt‐ och p . (2023) Plikt‐ och prövningsverkets årsredovisning 2022.

[aos17488-bib-0023] Rendahl, I. (1972) Prövning a synförmågan vid olika belysningsnivåer. Försvarsmedicin, 8, 86–91.

[aos17488-bib-0024] Rose, K.A. , Morgan, I.G. , Ip, J. , Kifley, A. , Huynh, S. , Smith, W. et al. (2008) Outdoor activity reduces the prevalence of myopia in children. Ophthalmology, 115, 1279–1285. 18294691 10.1016/j.ophtha.2007.12.019

[aos17488-bib-0025] Saw, S.‐M. , Tan, S.‐B. , Fung, D. , Chia, K.‐S. , Koh, D. , Tan, D.T.H. et al. (2004) IQ and the association with myopia in children. Investigative Ophthalmology & Visual Science, 45, 2943–2948. 15326105 10.1167/iovs.03-1296

[aos17488-bib-0026] Shapira, Y. , Mimouni, M. , Machluf, Y. , Chaiter, Y. , Saab, H. & Mezer, E. (2019) The increasing burden of myopia in Israel among young adults over a generation: analysis of predisposing factors. Ophthalmology, 126(12), 1617–1626. Available from: 10.1016/j.ophtha.2019.06.025 31474440

[aos17488-bib-0027] Thorisdottir, R.L. , Faxén, T. , Blohmé, J. , Sheikh, R. & Malmsjö, M. (2019) The impact of vision screening in preschool children on visual function in the Swedish adult population. Acta Ophthalmologica, 97, 793–797. 31127702 10.1111/aos.14147

[aos17488-bib-0028] Tscherning, M. (1882) Studier over myopiens ætiologi. København: C Myhres Boghandel.

[aos17488-bib-0029] Uhlin, D. , Lutteman, S. , Jennings, J.A.M. & Brautaset, R.L. (2009) Refractive trends in Swedish military recruits. Scandinavian Journal of Optometry and Visual Science, 2, 1–5.

[aos17488-bib-0030] Vannas, A.E. , Ying, G.‐S. , Stone, R.A. , Maguire, M.G. , Jormanainen, V. , Ying, G.‐.S. et al. (2003) Myopia and natural lighting extremes: risk factors in Finnish army conscripts. Acta Ophthalmologica Scandinavia, 81(6), 588–595. Available from: 10.1046/j.1395-3907.2003.0151.x 14641259

[aos17488-bib-0031] Vink, G. , Frank, L.E. , Pannekoek, J. & Van Buuren, S. (2014) Predictive mean matching imputation of semicontinuous variables. Statistica Neerlandica, 68, 61–90.

[aos17488-bib-0032] WHO . (2017) *WHO Report Myopia 2016: The impact of myopia and high myopia. University of New South Wales, Sydney, Australia 16–18 March 2015*. Report of the Joint World Health Organization–Brien Holden Vision Institute Global Scientific Meeting on Myopia, University of New South Wales, Sydney, Australia 16–18 March 2015.

[aos17488-bib-0033] Yang, L. , Vass, C. , Smith, L. , Juan, A. & Waldhör, T. (2020) Thirty‐five‐year trend in the prevalence of refractive error in Austrian conscripts based on 1.5 million participants. The British Journal of Ophthalmology, 104, 1338–1344. 32024654 10.1136/bjophthalmol-2019-315024

